# Uncovering oligodendrocyte enhancers that control *Cnp* expression

**DOI:** 10.1093/hmg/ddad141

**Published:** 2023-08-29

**Authors:** Chuandong Fan, Hongjoo An, Dongkyeong Kim, Yungki Park

**Affiliations:** Department of Biochemistry, Jacobs School of Medicine and Biomedical Sciences, Institute for Myelin and Glia Exploration, State University of New York at Buffalo, Buffalo, NY 14203, United States; Department of Biochemistry, Jacobs School of Medicine and Biomedical Sciences, Institute for Myelin and Glia Exploration, State University of New York at Buffalo, Buffalo, NY 14203, United States; Department of Biochemistry, Jacobs School of Medicine and Biomedical Sciences, Institute for Myelin and Glia Exploration, State University of New York at Buffalo, Buffalo, NY 14203, United States; Department of Molecular Pharmacology, Albert Einstein College of Medicine, Bronx, NY 10461, United States; Department of Biochemistry, Jacobs School of Medicine and Biomedical Sciences, Institute for Myelin and Glia Exploration, State University of New York at Buffalo, Buffalo, NY 14203, United States

**Keywords:** oligodendrocyte, enhancers, Cnp, transcription, myelin

## Abstract

Oligodendrocytes (OLs) produce myelin sheaths around axons in the central nervous system (CNS). Myelin accelerates the propagation of action potentials along axons and supports the integrity of axons. Impaired myelination has been linked to neurological and neuropsychiatric disorders. As a major component of CNS myelin, 2′,3′-cyclic nucleotide 3′-phosphodiesterase (CNP) plays an indispensable role in the axon-supportive function of myelin. Notably, this function requires a high-level expression of *CNP* in OLs, as evidenced by downregulated expression of *CNP* in mental disorders and animal models. Little is known about how *CNP* expression is regulated in OLs. Especially, OL enhancers that govern *CNP* remain elusive. We have recently developed a powerful method that links OL enhancers to target genes in a principled manner. Here, we applied it to *Cnp*, uncovering two OL enhancers for it (termed Cnp-E1 and Cnp-E2). Epigenome editing analysis revealed that Cnp-E1 and Cnp-E2 are dedicated to *Cnp*. ATAC-seq and ChIP-seq data show that Cnp-E1 and Cnp-E2 are conserved OL-specific enhancers. Single cell multi-omics data that jointly profile gene expression and chromatin accessibility suggest that Cnp-E2 plays an important role in *Cnp* expression in the early stage of OL differentiation while Cnp-E1 sustains it in mature OLs.

## Introduction

Oligodendrocytes (OLs) form myelin sheaths around axons in the central nervous system (CNS) [1]. Traditionally, myelin was considered an inert fatty insulator that accelerates the propagation of action potentials along axons [2]. It is now clear that myelin is a dynamic structure and plays an important role in the plasticity of the CNS that underlies learning and memory [3–7]. Myelin also provides metabolic and trophic support for axons [8–10]. Further, myelin fosters synaptogenesis [11] and mediates the effect of social experience on animal behavior [12,13].

2′,3′-cyclic nucleotide 3′-phosphodiesterase (Cnp) is one of the most abundant proteins in CNS myelin [14]. Although Cnp is dispensable for myelin assembly, it is indispensable for the integrity of axons and neural circuits [15]. In *Cnp* knockout mice (*Cnp*^−/−^), axoglial interactions, which underlie the domain-specific clustering of ion channels and cell adhesion molecules around the node of Ranvier, were disrupted [16]. These nodal defects were followed by axonal degeneration, microglial activation, and blood-brain barrier disruption [15]. Similar phenotypes were observed in humans and dogs [17,18]. At the molecular level, Cnp appears to counteract the membrane compaction by myelin basic protein (Mbp), establishing nano-channels in compact myelin sheaths that deliver crucial molecules and signals to axons [19].

Interestingly, *CNP* is one of the most downregulated OL genes in neuropsychiatric disorders [20,21], suggesting that sustained expression of *CNP* is required for the proper function of the CNS. Consistently, the brains of *Cnp* heterozygous knockout mice (*Cnp*^+/−^) exhibited microgliosis, astrogliosis, and axonal degeneration without overt demyelination upon aging [22]. These histological abnormalities were accompanied by behavioral changes, including anxiety, impaired social interaction, depression, and catatonia [22]. Taken together, *CNP* is haploinsufficient for the axon-supportive function of OL myelin, and a proper expression of *CNP* is essential for normal brain function.

In the CNS, *Cnp* is mainly expressed by OL lineage cells [23]. Its expression level is low in OL precursor cells (OPCs), but goes up dramatically as OPCs differentiate into OLs [23]. What drives the OL-specific expression of *Cnp* remains unknown. Simply put, the expression of a gene is regulated by upstream regulators acting on the gene’s enhancers (*cis*-regulatory DNA elements) [24,25]. To understand *Cnp* expression, thus, one needs to identify its enhancers and transcription factors acting on them. Logically, enhancer identification would come first because, without the knowledge of enhancers, it would not be feasible to identify transcription factors acting on them. A common feature of enhancers is that they can be found anywhere with regard to target genes—far upstream, near upstream, in gene body, near downstream, or far downstream. This made it a formidable challenge to map enhancers for a gene of interest. For this reason, OL enhancers that govern *Cnp* remain elusive. To tackle this fundamental issue, we developed an innovative method that links enhancers to target genes in a principled manner [26]. This new method has successfully uncovered enhancers for key OL genes such as *Myrf* [26], *Rgcc* [27], *Plp1* [28], and *Olig2* [29]. The present study applied it to *Cnp*, identifying two OL enhancers for it (termed Cnp-E1 and Cnp-E2).

## Results

### A systematic method to find OL enhancers for *Cnp*

**Figure 1:**
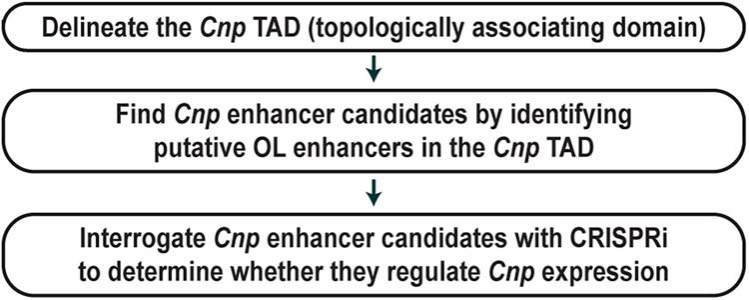
A systematic method to find OL enhancers for *Cnp*.

Our new method involves three steps, as described in Fig. 1. In the first step, we delineate the topologically associating domain (TAD) for *Cnp* by analyzing public Hi-C data. Chromatin interaction studies have shown that genes and their enhancers tend to be located within the same TAD, which is a fundamental unit of genome organization and function [30,31]. This leads us to the hypothesis that OL enhancers that control the expression of *Cnp* would be found in the TAD where *Cnp* is located. The internal detail of a TAD varies between cell types because it reflects cell type-specific gene-enhancer interactions. However, the boundary of a TAD is generally conserved between cell types and species [30,32], which allowed us to define OL gene TADs from non-OL Hi-C data [26–29]. Recently, human OL Hi-C data became available [33], facilitating OL gene TAD analysis. Second, all putative OL enhancers within the *Cnp* TAD are identified. Since they are in the same TAD as *Cnp*, they are considered potential candidates for *Cnp* enhancers. Our previous study generated a genome-wide map of putative OL enhancers by integrating OL ChIP-seq data [26]. This genome-wide map is compared with the *Cnp* TAD to identify all putative OL enhancers within the *Cnp* TAD. In summary, the first and second steps narrow down the enhancer search space from the entire genome to a few discrete loci. In the third step, *Cnp* enhancer candidates are silenced by CRISPRi, a cutting-edge epigenome editing technique [34–38], to determine whether they regulate *Cnp* expression.

### TAD analysis for *Cnp*

**Figure 2:**
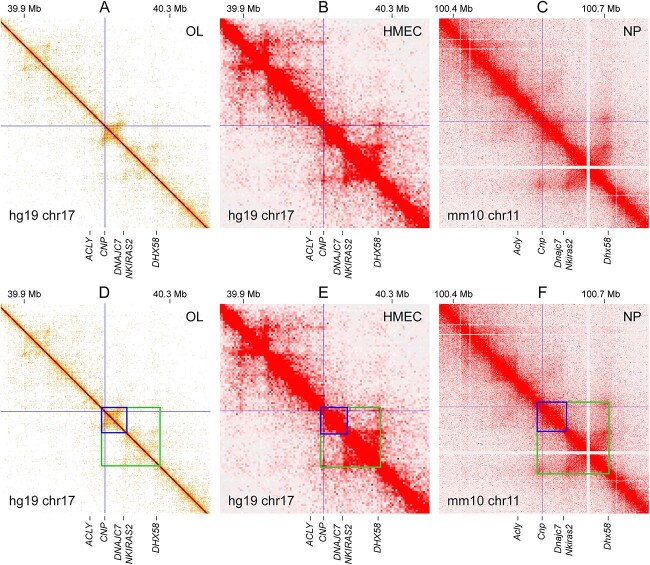
TAD analysis for *Cnp*. Public Hi-C data for human OLs, human mammary epithelial cells (HMECs), and mouse neural progenitors (NPs) were analyzed. On the diagonal is the genome. Off the diagonal, the interaction strength between two loci is indicated by color tone. White means no interaction, and orange (panels A and D) and red (the other four panels) the strongest interaction. The *CNP*/*Cnp* promoter locations are marked by thin crossing lines. The *CNP*/*Cnp* TAD is marked by a blue box in panels D–F. A larger TAD containing it is marked by a green box. Please see Materials and Methods for the sources of these data. These figures were generated by Juicebox [78,79] and HiGlass [80]. Magnified versions of these figures are available in Supplementary Material, Fig. S2.

To define the *CNP*/*Cnp* TAD, we examined public Hi-C data for human and mouse cell types [31,33,39]. In each panel of Fig. 2, the position of the *CNP*/*Cnp* promoter is shown by thin crossing lines. The diagonal represents the genome. Off the diagonal, the interaction strength between two loci is indicated by color tone. White means no interaction. Orange (panels A and D) and red (the other four panels) mean the strongest interaction. The OL Hi-C data (Fig. 2A) reveal that *CNP* is located in a TAD (marked by a blue box in Fig. 2D) that is part of a larger TAD (marked by a green box in Fig. 2D). Remarkably, this TAD organization is conserved across cell types and species, as judged by the locations of the TAD boundaries and nearby genes (human mammary epithelial cells (HMEC) in Fig. 2B and E and mouse neural progenitors (NP) in Fig. 2C and F). The *CNP* TAD spans about 60 Kb (the blue box in Fig. 2D). Its strong evolutionary conservation suggests that critical OL enhancers for *CNP* would be found within it.

### Identification and CRISPRi interrogation of 3 *Cnp* enhancer candidates

**Figure 3:**
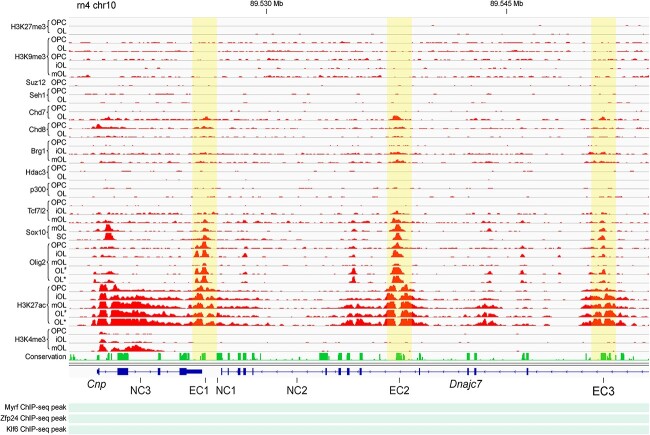
Three *Cnp* ECs. Rat OL ChIP-seq data were compiled for them. Also shown are the locations of three negative control (NC) regions for epigenome editing analysis. Of note, the region shown here is fully contained in the *Cnp* TAD demarcated in Fig. 2. iOL: immature OL. mOL: mature OL. SC: spinal cord. OL^#^ and OL^*^: OLs treated with vehicle and lysophosphatidylcholine, respectively. For the Myrf ChIP-seq data, only peak locations are shown because the raw data is not available. The mouse Zfp24 and Klf6 ChIP-seq data were mapped to the rat genome by LiftOver. Please see Materials and Methods for the sources of these data. This figure was generated by the IGV browser [81].

We compared our genome-wide map of putative OL enhancers [26] with the *Cnp* TAD. As a result, we identified three putative OL enhancers within it, referred to as *Cnp* enhancer candidates (EC1, 2, and 3, highlighted in yellow in Fig. 3). The ranking of these enhancer candidates was based on the strength of the underlying data shown in Fig. 3, with EC1 being the strongest and EC3 being the weakest. Computational details behind this data integration were described in our previous paper [26]. All three ECs are prominently marked by H3K27ac peak-valley-peaks (Fig. 3), an epigenetic feature associated with active enhancers [40]. They lack H3K4me3 (a promoter mark), further supporting their enhancer identity. They are bound by key OL transcription factors, namely Olig2, Sox10, and Tcf7l2, suggesting their potential involvement in OL-specific gene regulation. However, they are not bound by Myrf, Zfp24, and Klf6 (Fig. 3). Since our goal was to find conserved OL enhancers for *Cnp*, we did not pursue putative rat-specific OL enhancers around EC2 and EC3. Our criteria for calling putative OL enhancers were quite lenient. Thus, it remains uncertain whether the three *Cnp* ECs truly function as OL enhancers. Additionally, even if they do, it is not guaranteed that they control *Cnp* expression. These uncertainties highlight the need for further investigation, including the use of CRISPRi and other methods to interrogate the functionality of these enhancer candidates.

CRISPRi is a cutting-edge epigenome editing technique [34–38]. In CRISPRi, dCas9-KRAB, a fusion protein between a nuclease-null Cas9 (dCas9) and a KRAB domain, is targeted to a specific locus by guide RNAs (gRNAs). When targeted to a promoter, dCas9-KRAB silences it by inducing trimethylation of H3K9 (K9 of histone 3) [36]. When targeted to an enhancer, dCas9-KRAB silences it by the same mechanism, which in turn downregulates its target genes. This is how one can map enhancers to target genes by CRISPRi.

To silence the three *Cnp* ECs, dCas9-KRAB was delivered to them by a pool of 4 gRNAs (G1-4) in Oli-neu cells, a widely used OL cell line [41]. Specifically, gRNAs were cloned into an in-house piggyBac-based plasmid and inserted into the genome of Oli-neu cells that express dCas9-KRAB in a doxycycline-dependent manner (Supplementary Material, Fig. S1, see Materials and Methods). In resulting cell lines, gRNAs were expressed constitutively while the expression of dCas9-KRAB was induced by doxycycline. In addition, two Oli-neu cell lines were generated using non-targeting gRNAs called Scr1 and Scr2. These scrambled gRNAs do not have a specific target in the mouse genome and were used as negative controls to assess the effects of the experimental manipulation. One Oli-neu cell line was produced as a positive control. In this case, dCas9-KRAB was targeted to the *Cnp* promoter by a pool of four gRNAs. Since the three *Cnp* ECs are found in the vicinity of *Cnp*, knocking them down by CRISPRi may also silence the *Cnp* promoter non-specifically. In order to rule out this possibility, three negative control regions around the three *Cnp* ECs (NC1-3 in Fig. 3) were also targeted by dCas9-KRAB with a pool of four gRNAs. Oli-neu cell lines were cultured in the differentiation condition for 2 days in the presence of doxycycline to induce *Cnp* expression and execute CRISPRi epigenome editing. Then, RNA was extracted, and RT-qPCR performed to determine *Cnp* expression.

**Figure 4:**
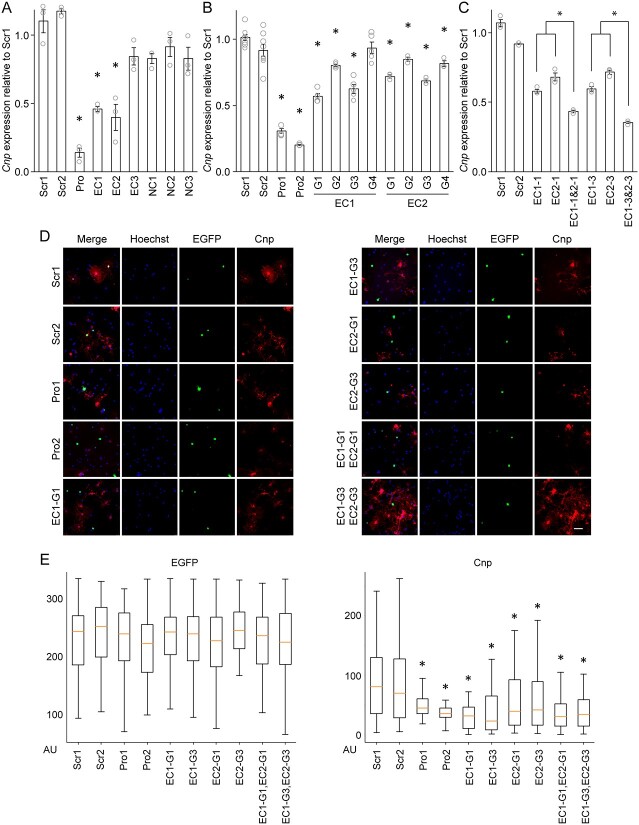
CRISPRi interrogation of *Cnp* ECs. (A) RT-qPCR analysis of *Cnp* expression in Oli-neu cells after CRISPRi knockdown of *Cnp* ECs. Shown are data points (biological replicates) and their mean and standard error. ^*^p < 4.17 × 10^−2^ by Student’s *t* test with Bonferroni correction. (B) Same as panel A, except that dCas9-KRAB was brought to each target by individual gRNAs. Shown are data points (biological replicates) and their mean and standard error. ^*^p < 3.01 × 10^−2^ by Student’s *t* test with Bonferroni correction. (C) Effect of simultaneously silencing EC1 and EC2 on the expression of *Cnp* in Oli-neu cells. Shown are data points (biological replicates) and their mean and standard error. ^*^p < 4.33 × 10^−3^ by Student’s *t* test with Bonferroni correction. (D) Quantitative immunofluorescence of Cnp expression in mouse OLs after CRISPRi knockdown of EC1 and EC2. Shown are representative images of the 10 samples. Scale bar, 50 μm. Zoomed in images are available in Supplementary Material, Fig. S3. (E) EGFP and Cnp signals were quantified for individual cells by CellProfiler and compared among the samples. The number of EGFP-positive cells analyzed is as follows: Scr1 (84), Scr2 (106), Pro1 (86), Pro2 (86), EC1-G1 (62), EC1-G3 (61), EC2-G1 (52), EC2-G3 (81), EC1&2-G1 (84), and EC1&2-G3 (85). ^*^p < 1.86 × 10^−2^ by Student’s *t* test with Bonferroni correction for comparison with Scr1. AU: arbitrary unit.

The expression level of *Cnp* was comparable between Scr1 and Scr2 (Scr1 and Scr2 in Fig. 4A). It dropped dramatically when dCas9-KRAB was targeted to the *Cnp* promoter by the pool of 4 gRNAs (Pro in Fig. 4A). Silencing EC1 and EC2 by CRISPRi also led to a significant decrease in *Cnp* expression (Fig. 4A). The specificity of these results was demonstrated by the observation that targeting dCas9-KRAB to EC3 and the three NCs did not affect the expression of *Cnp*. To exclude off-target effects of gRNAs, we repeated the CRISPRi experiment for EC1 and EC2, this time using individual gRNAs (G1-4 for EC1 and EC2 in Fig. 4B). Two individual gRNAs for the *Cnp* promoter (Pro1 and Pro2) were used as positive controls. Multiple EC1 and EC2 gRNAs led to a significant knockdown of *Cnp* (Fig. 4B), ruling out off-target effects. As expected, the effects of individual gRNAs were not as strong as those of pooled gRNAs. Silencing both EC1 and EC2 by either EC1-G1 & EC2-G1 or EC1-G3 & EC2-G3 suppressed *Cnp* expression to a greater extent (Fig. 4C). These results demonstrate that silencing of EC1 and EC2 is sufficient to reduce *Cnp* expression in Oli-neu cells.

Although Oli-neu cells are a good model for OL lineage cells, they are not OL lineage cells. Findings from Oli-neu cells have to be validated with primary OLs to ensure their physiological relevance. To test EC1 and EC2 in primary OLs, the CRISPRi experiment was repeated with mouse OPCs [42,43]. Challenges with transfection efficiency and the infeasibility of drug selection made it impossible to perform RT-qPCR for them. Instead, we had to rely on quantitative immunofluorescence. In this experiment, we determine the effect of silencing enhancers on gene expression in individual cells by single-cell quantitative immunofluorescence. Thus, high transfection efficiency is not required. A plasmid of dox-inducible dCas9-KRAB and EGFP was transfected into mouse OPCs, together with gRNA plasmids. Transfected OPCs were cultured in the differentiation condition for 2 days to induce their differentiation into OLs and *Cnp* expression. They were stained for EGFP (identifying transfected cells) and Cnp. As above, Scr1 and Scr2 were used as negative controls, and Pro1 and Pro2 as positive controls. For EC1 and EC2, two gRNAs were used (G1 and G3). We also included two EC1 and EC2 gRNA combinations shown in Fig. 4C. For each of the 10 samples (Fig. 4D), at least 50 pictures were taken. Signals from three fluorescence channels (Hoechst, EGFP, and Cnp) were quantified for individual OLs by CellProfiler [44]. This quantitative single-cell image analysis revealed that while the CRISPRi components (dCas9-KRAB and gRNA) were expressed at comparable levels for EGFP+ cells across the 10 samples (Fig. 4E), Cnp signals were much lower when dCas9-KRAB was targeted to the *Cnp* promoter, EC1, EC2, or EC1 and EC2 (Fig. 4E). Overall, these results demonstrate that EC1 and EC2 promote *Cnp* expression in primary OLs.

### EC1 and EC2 are dedicated to *Cnp*

**Figure 5:**
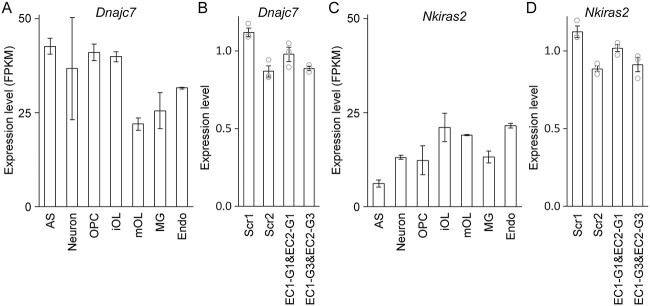
Two genes in the vicinity of the *CNP/Cnp* TAD. (A) Expression profile of *Dnajc7* in brain cell types [23]. AS: astrocytes. iOL: immature OLs. mOL: mature OLs. MG: microglia. Endo: endothelial cells. (B) RT-qPCR analysis of *Dnajc7* expression in Oli-neu cells after CRISPRi knockdown of EC1 and EC2. Shown are data points (biological replicates) and their mean and standard error. (C) Expression profile of *Nkiras2* in brain cell types [23]. (D) RT-qPCR analysis of *Nkiras2* expression in Oli-neu cells after CRISPRi knockdown of EC1 and EC2. Shown are data points (biological replicates) and their mean and standard error.

*Dnajc7* and *Nkiras2* lie on the border of the *Cnp* TAD (Fig. 2). Brain RNA-seq data indicate that they are expressed in various brain cell types [23], including OL lineage cells (Fig. 5A and C). Since enhancers can control multiple genes [37,38], we wondered whether EC1 and EC2 regulate *Dnajc7* and *Nkiras2* as well. To address this, the RNA samples of Fig. 4C were reanalyzed by RT-qPCR for *Dnajc7* and *Nkiras2*. Simultaneous knockdown of EC1 and EC2 by CRISPRi did not affect their expression (Fig. 5B and D), highlighting the specificity of EC1 and EC2 toward *Cnp*.

### Cell type specificity of Cnp-E1 and Cnp-E2

**Figure 6:**
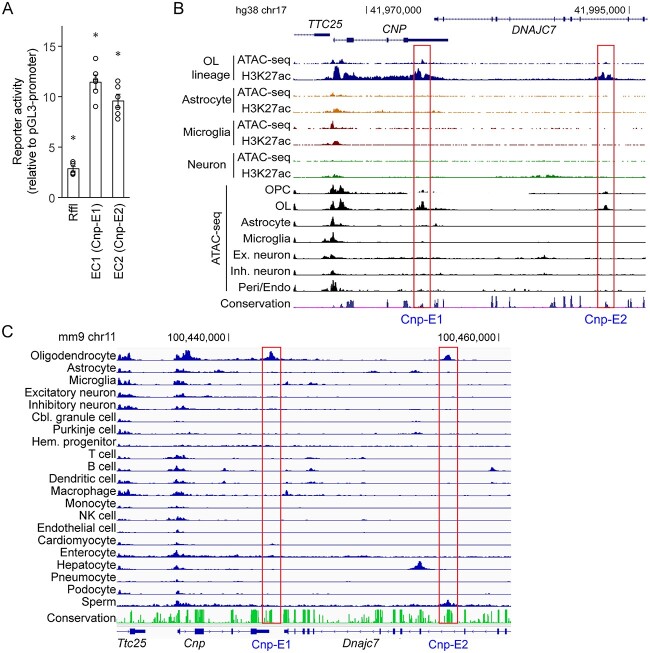
Cell type specificity of EC1 and EC2. (A) Luciferase assay results for EC1 and EC2 that were transfected into mouse OPCs cultured in the differentiation conditions for 2 days. Shown are data points (biological replicates) and their mean and standard error. ^*^p < 1.45 × 10^−3^ by Student’s *t* test with Bonferroni correction. EC1 and EC2 are henceforth referred to as Cnp-E1 and Cnp-E2, respectively. (B) Human brain cell type-specific ChIP-seq and ATAC-seq data for Cnp-E1 and Cnp-E2. (C) Mouse single-cell ATAC-seq data for Cnp-E1 and Cnp-E2. Please see Materials and Methods for the sources of these data.

EC1 and EC2 exhibit epigenetic features of enhancers (Fig. 3). Consistently, the CRISPRi experiment showed that they activate *Cnp* expression. These observations suggest that they work as OL enhancers. To directly test this hypothesis, we performed a luciferase assay. EC1 and EC2 were cloned into pGL3-promoter and transfected into mouse OPCs. Rffl, an OL-specific enhancer [45–47], was included as a positive control. pGL3-promoter (empty vector) was used to estimate the baseline. Transfected OPCs were cultured in the differentiation condition for 2 days to induce their differentiation into OLs. Rffl significantly elevated the reporter activity of pGL3-promoter (Fig. 6A), validating our experimental condition. Under this condition, EC1 and EC2 also exhibited such activity (Fig. 6A), and they were more powerful than Rffl in doing so (*p* value < 2.62 × 10^−6^ by *t* test and adjusted by the Bonferroni correction). Having shown that EC1 and EC2 work as OL enhancers that activate *Cnp* expression, they will henceforth be referred to as Cnp-E1 and Cnp-E2, respectively.

We analyzed public datasets to elucidate the cell type specificity of Cnp-E1 and Cnp-E2. First, we looked up the human brain single-nucleus ATAC-seq data from Swarup and colleagues [48]. We calibrated it with the loci of *GAPDH* and *ACTB* for a quantitative comparison of peak heights among different cell types. The single-nucleus ATAC-seq data reveal that Cnp-E1 and Cnp-E2 are open only in OL lineage cells (OPC and OL, Fig. 6B). Second, we explored the human brain cell type-specific ChIP-seq and ATAC-seq data from Glass and co-workers [49]. We calibrated these data in the same way. Cnp-E1 and Cnp-E2 coincide with OL-specific H3K27ac peak-valley-peaks and ATAC-seq peaks (Fig. 6B), which agree with the data from Swarup and colleagues. Third, to check the specificity of Cnp-E1 and Cnp-E2 more broadly, we inspected the H3K27ac ChIP-seq data from the NIH Roadmap Epigenomics Project [50], which were calibrated in the same manner. It shows that Cnp-E1 and Cnp-E2 are active only in the brain tissues (Supplementary Material, Fig. S4), supporting their OL specificity. Finally, we examined the mouse single-cell ATAC-seq data from Shendure and colleagues, which were clustered into 21 cell types and calibrated in the same way [51]. This dataset does not have data for OPCs. It points out that Cnp-E1 and Cnp-E2 are specific to OLs, with a possible exception that Cnp-E2 may also be active in sperms (Fig. 6C). Collectively, we conclude that Cnp-E1 and Cnp-E2 are evolutionarily conserved enhancers that are mostly specific to OL lineage cells.

### Temporal dynamics of Cnp-E1 and Cnp-E2

**Figure 7:**
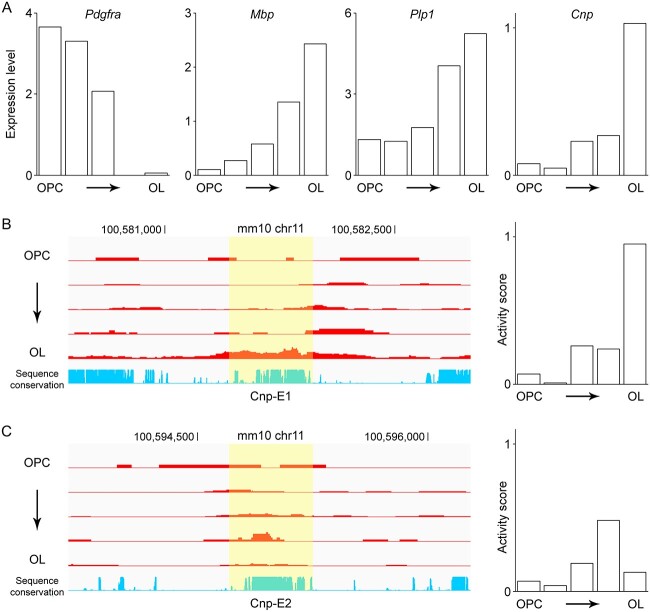
Temporal dynamics of Cnp-E1 and Cnp-E2. (A) Expression dynamics of OPC and OL marker genes during OL lineage progression, as defined by the monocle pseudotime analysis of the ISSAAC-seq mouse brain data. (B) Corresponding accessibility dynamics of Cnp-E1. Left: the ISSAAC-seq data visualized by the IGV browser. The y-axis represents the magnitude of the ATAC-seq signal, which was normalized by the number of cells in each group. Right: Cnp-E1 activity quantification. (C) Corresponding accessibility dynamics of Cnp-E2. Left: the ISSAAC-seq data visualized by the IGV browser. The y-axis represents the magnitude of the ATAC-seq signal, which was normalized by the number of cells in each group. Right: Cnp-E2 activity quantification. Please see Materials and Methods for the sources of these data.

ISSAAC-seq is an innovative method that simultaneously determines gene expression and chromatin accessibility of individual cells [52]. In other words, it performs single cell RNA-seq and single cell ATAC-seq for same cells in parallel. We analyzed a public ISSAAC-seq mouse brain dataset to examine the temporal dynamics of Cnp-E1 and Cnp-E2 during OL development and how it is correlated with *Cnp* expression. A clustering analysis of the single cell RNA-seq data identified 256 OL lineage cells. We carried out pseudotime analysis for them to order them along the lineage (i.e. from OPCs to OLs). Expression dynamics of OPC and OL marker genes validated our pseudotime analysis (Fig. 7A). The expression of *Cnp* peaks at the last stage (Fig. 7A). Then, we looked up the single cell ATAC-seq data for the chromatin accessibility of Cnp-E1 and Cnp-E2 for the 256 OL lineage cells. The accessibility profile of Cnp-E1 mirrors the expression pattern of *Cnp* (Fig. 7B). In contrast, Cnp-E2 opens at an earlier stage and appears to close at the last stage (Fig. 7C). This profile is consistent with the brain cell type-specific ATAC-seq and ChIP-seq data showing that the enhancer-associated features of Cnp-E1 are more pronounced than those of Cnp-E2 in OLs (Fig. 6B). These data suggest that Cnp-E2 plays an important role in *Cnp* expression in the early stage of OL differentiation while Cnp-E1 sustains it in mature OLs.

## Discussion

Our study reports the identification of long-sought OL enhancers for *Cnp* (Cnp-E1 and Cnp-E2). They are conserved across species, as evidenced by sequence conservation and enhancer-associated epigenetic marks. Therefore, even though we performed epigenome editing analysis in the mouse genome, we expect Cnp-E1 and Cnp-E2 to play a similar role in human OLs.

To comprehend the expression of *Cnp* in OLs, it is essential to identify OL enhancers responsible for its regulation and upstream transcription factors that act on them. Logically, the initial step revolves around the identification of *Cnp* enhancers, as without such knowledge, it becomes challenging to determine transcription factors that influence them. Our discovery of Cnp-E1 and Cnp-E2 provides an opportunity to systematically uncover upstream regulators of *Cnp*. If a transcription factor activates *Cnp* expression by interacting with Cnp-E1 or Cnp-E2, depleting that factor would decrease the enhancer activity of Cnp-E1 or Cnp-E2. Conversely, if a transcription factor represses *Cnp* expression by acting on Cnp-E1 or Cnp-E2, its depletion would lead to an increase in the enhancer activity of Cnp-E1 or Cnp-E2. Changes in the enhancer activity of Cnp-E1 and Cnp-E2 can easily be read out by luciferase assay. Capitalizing on this powerful framework, together with our in-house library of CRISPRi gRNAs, we plan to perform a large-scale CRISPRi screen where each transcription factor is knocked down by CRISPRi and its impact on the enhancer activity of Cnp-E1 and Cnp-E2 measured by luciferase assay. Once upstream regulators are identified from the CRISPRi screen, we would classify them into direct and indirect ones by DNA pulldown assay. Direct upstream regulators control *Cnp* expression by binding to Cnp-E1 and/or Cnp-E2. Indirect ones do so without binding. They are thought to affect direct regulators or other indirect ones.

To get a glimpse of the repertoire of transcription factors that may regulate *Cnp* expression via Cnp-E1 and Cnp-E2, we performed a bioinformatics analysis. By using FIMO [53], together with HOCOMOCO [54], we scanned the DNA sequences of Cnp-E1 and Cnp-E2 and identified high-confidence motif matches (Supplementary Material, Table S2). Of note, transcription factors that belong to same families often recognize similar motifs, and it is quite difficult to predict which member of a family binds just from a motif match. For this reason, we discuss motif match results in terms of transcription factor families rather than individual members. Several intriguing hypotheses emerge from the motif analysis. First, nuclear factor I family members are predicted to bind to Cnp-E1 and Cnp-E2. Given the previous report that nuclear factor IA hinders OL maturation [55], this finding suggests that it may do so by repressing *Cnp* expression via Cnp-E1 and Cnp-E2. Second, there are significant motif matches in both enhancers for Sox (SRY-related HMG-box) family members. They may play a dynamic role in *Cnp* expression during the OL lineage progression. For example, some members may repress it in OPCs by acting on Cnp-E1 and Cnp-E2. As OPCs differentiate into OLs, they may be replaced by activating members (e.g. Sox8 and Sox10) for the expression of *Cnp*. This type of role switching may also be played by Krüppel-like factors. Finally, several transcription factor families with no known role in OL biology are predicted to bind to Cnp-E1 and Cnp-E2, suggesting that there are still more transcription factors to be discovered with regard to OL development.

Identifying upstream regulators of *Cnp* and mapping the binding sites of direct ones within Cnp-E1 and Cnp-E2 are significant areas of interest. A proper expression of *CNP* is essential for normal brain function, as evidenced by the finding that *CNP* is one of the most downregulated OL genes in neuropsychiatric disorders. If the expression levels of *CNP* upstream regulators change or mutations occur in the binding sites of direct ones, the expression of *CNP* may be altered, potentially disrupting the axon-supportive function of myelin. This knowledge would contribute to unraveling the molecular mechanisms underlying CNP-associated disorders and may pave the way for therapeutic interventions.

Another intriguing aspect of Cnp-E1 and Cnp-E2 is their distinct activity patterns during the OL lineage, as suggested by the ISSAAC-seq mouse brain data [52]. Cnp-E2 is transiently active in early differentiating OLs. This predicts that the deletion of Cnp-E2 impedes *Cnp* expression during the early stages of OL differentiation, while it is unlikely to have a significant impact on *Cnp* expression in mature OLs. On the contrary, Cnp-E1 remains inactive throughout most stages and becomes active only at the final stage. Thus, Cnp-E1 is predicted to sustain the expression of *Cnp* in mature OLs, playing a crucial role in the life-long maintenance of *Cnp* expression. To test these hypotheses, we plan to generate mice where Cnp-E1 and Cnp-E2 are flanked by loxP sequences for conditional knockout. These investigations would shed light on the specific contributions of Cnp-E1 and Cnp-E2 to the regulation of *Cnp* expression during OL development and function.

## Materials and methods

### Animal procedures, tissue harvest, and cell culture

Animal husbandry was carried out in accordance with a protocol approved by Institutional Animal Care and Use Committee. OPCs were purified from mouse pups by immunopanning [42,43]. The original protocol for mouse OPCs [42] did not work in our hands. Thus, we made one significant change. Instead of a positive selection with anti-Pdgfrα antibody [42], we performed a negative selection with anti-O1 antibody and a positive selection with anti-O4 antibody, as described for rat OPCs [43]. Upon immunopanning, we amplified OPCs by culturing them with PDGF-AA for 6–7 days. During this amplification period, non-OPCs are diluted out, resulting in a pure population of OPCs. A more detailed protocol is available upon request. OPCs and Oli-neu cells [41] were kept in a proliferative condition by supplementing the Sato media [43] with PDGF-AA (5 ng/ml), NT-3 (0.5 ng/ml), CNTF (5 ng/ml), and B-27 (1:200 dilution). To induce their differentiation, PDGF-AA was omitted, and T3 was added (40 ng/ml). For Oli-neu cells, PD 174265 (1 nM) was also added to inhibit EGFR tyrosine kinase. Cells were maintained in a humidified 7% CO_2_ incubator at 37°C. All transfections were carried out by using Lipofectamine 2000 as per the manufacturer’s instructions.

### CRISPRi constructs: dCas9-KRAB

Two dCas9-KRAB constructs were generated. First, “dCas9-KRAB-RB” (Supplementary Material, Fig. S1), a doxycycline-inducible dCas9-KRAB that can be integrated into the genome of Oli-neu cells, was generated by modifying pAAVS1-NDi-CRISPRi (Addgene 73497) as follows. First, an RB (RFP and blasticidin resistance) cassette was fused to the rtTA via P2A. Second, the inverted terminal repeats (ITRs) recognized by SB100X (Addgene 34879) [56] were inserted. dCas9-KRAB-RB was used to generate stable cell lines (see below). Second, “dCas9-KRAB-GP” was generated in the same way except that an GP (GFP and puromycin resistance) cassette was fused to the rtTA instead of the RB cassette. dCas9-KRAB-GP was used for quantitative immunofluorescence experiments. Sequence information for dCas9-KRAB-RB and dCas9-KRAB-GP was verified by Sanger sequencing.

### CRISPRi constructs: guide RNAs

A guide RNA (gRNA)-expressing construct, “PB-GP-U6” (Supplementary Material, Fig. S1), was generated as follows. First, the content of PB-CA (Addgene 20960) was replaced by the sgRNA scaffold of lentiCRISPR v2 (Addgene 52961). Second, the GP cassette was inserted. gRNAs cloned into PB-GP-U6 were used to generate stable cell lines and for quantitative immunofluorescence experiments. The sequences of gRNAs used for the current study are in Supplementary Material, Table S1. Sequence information for all gRNA constructs was confirmed by Sanger sequencing.

### Stable cell line generation

dCas9-KRAB-RB and SB100X were co-transfected into Oli-neu cells [41]. SB100X [56], a hyperactive transposase, recognizes the ITRs of dCas9-KRAB-RB and integrates whatever flanked by the ITRs into the genome (Supplementary Material, Fig. S1). Hence, cells that proliferate in the presence of blasticidin express dCas9-KRAB in a doxycycline-dependent manner. Similarly, PB-GP-U6 and hypBase [57] were co-transfected into Oli-neu cells. hypBase, a hyperactive transposase, recognizes the ITRs of PB-GP-U6 and inserts whatever flanked by the ITRs into the genome (Supplementary Material, Fig. S1). Thus, cells that proliferate in the presence of puromycin constitutively express gRNAs. Of note, the SB100X and hypBase plasmids themselves are not inserted into the genome. They are diluted out during cell proliferation. The hypBase plasmid was generously provided by Breunig [58]. To generate stable cell lines for single gRNAs, four plasmids (dCas9-KRAB-RB, SB100X, PB-GP-U6, hypBase) were co-transfected, and transfected cells were subjected to drug selection with blasticidin and puromycin. To generate stable cell lines for pooled gRNAs, seven plasmids were co-transfected (dCas9-KRAB-RB, SB100X, 4 PB-GP-U6 plasmids, hypBase), and transfected cells were subjected to the same drug selection. The transposon-assisted genomic integration of plasmids is highly effective. Together with the fact that Oli-neu cells can easily be killed by blasticidin and puromycin, it allows us to produce desired cell lines in a week [26–29,59]. There is no need to grow single cell clones and check gene expression for them, as when generating stable cell lines via random genomic insertion. Oli-neu cells were cultured in the proliferation condition during the drug selection process. Once it is over, Oli-neu cells were treated with doxycycline (1 ug/ml) for 2 days in the differentiation condition to induce *Cnp* expression and execute CRISPRi epigenome editing. Then, RNA was harvested for RT-qPCR.

### RT-qPCR

Total RNA was purified by using Trizol (ThermoFisher 15596026), and cDNA synthesized by the SuperScript First-Strand kit (Invitrogen 11904-018). Quantitative PCR was performed on C1000 Thermal Cycler with CFX96 optical reaction module (Bio-rad). *Gapdh* was used as a loading control. Each PCR reaction contained 2 μl of cDNA, 5 μl of the iTaq Universal SYBR Green Supermix (Bio-rad 1725124), and 500 nM of forward and reverse primers. The primer sequences are as follows.

*Gapdh* (forward): GGT GAA GGT CGG TGT GAA CGG.

*Gapdh* (reverse): CTG GAA CAT GTA GAC CAT GTA GTT GAG G.

*Cnp* (forward): GGG AAT CAC AAG GCC TTC AAG AAA G.

*Cnp* (reverse): CAG CAC ACC TGG AGG TCT C.

*Dnajc7* (forward): CAC AGC AGG AGT TCA AGA ACG.

*Dnajc7* (reverse): CAT GCA GAA AAC AAC CTT CCG G.

*Nkiras2* (forward): CCA TGT CGT GGG TTC TGA GAT G.

*Nkiras2* (reverse): CCC CGT GTA TCA TAG AAA CGC A.

### Immunofluorescence

Cells were fixed with 4% formaldehyde and permeabilized with 0.1% Triton X-100. Upon blocking with 1% BSA, they were incubated with primary antibodies diluted in blocking buffer at 4°C overnight, followed by incubation with fluorochrome-conjugated secondary antibodies. Nuclei were stained with Hoechst 33342 (Invitrogen). Fluorescence was visualized with Leica DMi8 microscope with ORCA-Flash4.0 sCMOS camera. Reagents used for immunofluorescence are as follows: Cnp (Abcam ab6319), EGFP (BioLegend 338001), donkey anti-Mouse IgG, Alexa Fluor 594 (ThermoFisher A21203), and donkey anti-Rat IgG, Alexa Fluor 488 (ThermoFisher A21208).

### Luciferase assay

Cnp-E1 (mm10 chr11:100581624–100582086) and Cnp-E2 (mm10 chr11:100594705–100595099) were cloned into pGL3-promoter. Luciferase assay was performed by using the Firefly & Renilla Luciferase Single Tube Assay Kit from Biotium as per the manufacturer’s instructions. pRL-TK was used as an internal control. The ratio between firefly and renilla luciferase activities was taken as the reporter activity.

### OL ChIP-seq data

OL ChIP-seq data were downloaded from the Sequence Read Archive (SRA, https://www.ncbi.nlm.nih.gov/sra): GSE42454 (H3K9me3, Brg1, Olig2, H3K27ac, H3K4me3) [60], GSE72727 (Chd7, Sox10) [61], GSE119816 (Seh1) [62], GSE76411 (Hdac3, p300) [63], GSE82165 (Suz12) [64], GSE65119 (Tcf7l2) [65], GSE84011 (Olig2, H3K27ac) [66], GSE64703 (Sox10) [67], GSE107919 (Chd7, Chd8) [68], GSE101535 (Zfp24) [69], and GSE79243 (Klf6) [70]. The Myrf ChIP-seq data were downloaded from the journal website [45]. H3K27me3 and H3K9me3 data were kindly provided by Dr Patrizia Casaccia [71]. ChIP-seq reads were mapped to rn4 by Bowtie2 [72], and peaks called by MACS2 [73].

### Public genomic data

The Hi-C data for human mammary epithelial cells and mouse neural progenitors [31,39] were downloaded from the 4DN Web Portal (https://4dnucleome.org). The human OL Hi-C data [33] were downloaded from a public box directory at https://github.com/dixonlab/scm3C-seq. Human brain single-nucleus ATAC-seq data were downloaded from the Swarup laboratory website [48]. Human brain cell type-specific ATAC-seq and ChIP-seq data from Glass and coworkers [49] are available at https://genome.ucsc.edu/s/nottalexi/glassLab_BrainCellTypes_hg19. The H3K27ac ChIP-seq data from the Roadmap Epigenomics Project [50] were visualized by the WASHU Epigenome Browser. Mouse single-cell ATAC-seq data [51] were downloaded from the Shendure laboratory website (https://atlas.brotmanbaty.org).

### Mouse brain ISSAAC-seq data

As described in the original paper [52], the single cell RNA-seq reads were mapped to the mouse genome (mm10) by STAR [74]. The single cell ATAC-seq reads were mapped to mm10 by Cell Ranger ATAC (10× Genomics). Mapped reads were analyzed by Seurat [75] and Signac [76]. Pseudotime analysis was carried out by Monocle [77]. Low-quality cells were excluded from the analysis—those with more than 1% of mitochondrial genes, those with less than 2% of transcriptional start site enrichment, those with greater than 4% of nucleosome signal patterns, and those with greater than 5% of blacklist region mapping. Altogether, 256 OL lineage cells were analyzed.

##  

*Conflict of interest statement*: None declared.

## Funding

This work was supported by the National Institutes of Health [R21NS102558, R21NS112608, R21NS114476, and R21NS123775 to Y.P.].

## Supplementary Material

2023-05-30_Supplemental_Data_ddad141
