# Supplementary material for: Uncovering oligodendrocyte enhancers that control Cnp expression
Source: Hum Mol Genet. 2023 Aug 29;32(23):3225–36. doi: 10.1093/hmg/ddad141 (PMC10656706; doi:10.1093/hmg/ddad141)
Supplement: 2023-05-30_Supplemental_Data_ddad141 [file 2023-05-30_supplemental_data_ddad141.docx]

**Supplemental Data**

**Uncovering oligodendrocyte enhancers that control *Cnp* expression**

Chuandong Fan^1,#^, Hongjoo An^1,#^, Dongkyeong Kim^1,2^, and Yungki Park^1,^*

^1^Institute for Myelin and Glia Exploration, Department of Biochemistry, Jacobs School of Medicine and Biomedical Sciences, State University of New York at Buffalo, Buffalo, NY 14203, USA

^2^Present address: Department of Molecular Pharmacology, Albert Einstein College of Medicine, Bronx, NY 10461, USA

^#^Equal contribution

*To whom correspondence should be addressed. Tel: 1-716-881-7579; Fax: 1-716-849-6651; Email: [yungkipa@buffalo.edu](mailto:yungkipa@buffalo.edu)

**Figure S1. Transposon-based plasmids used to generate stable cell lines**


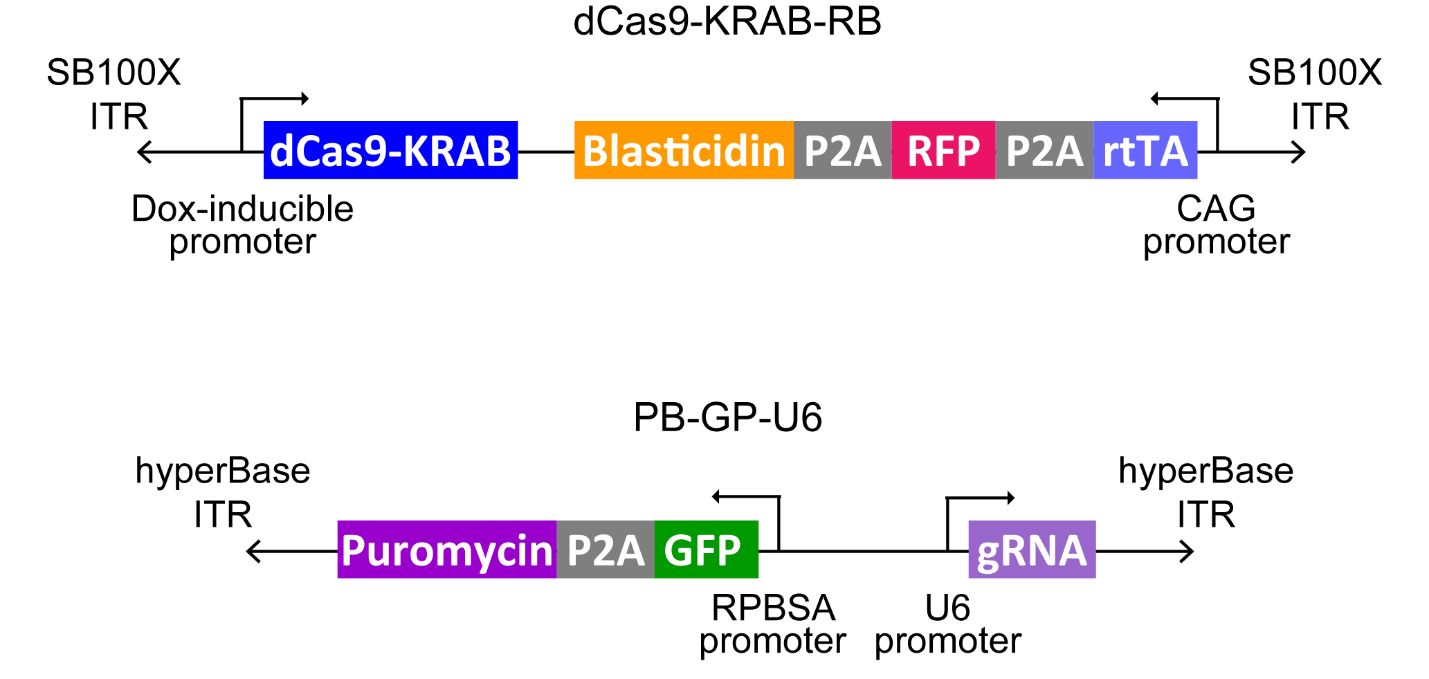


**Figure S2. Magnified Hi-C data**


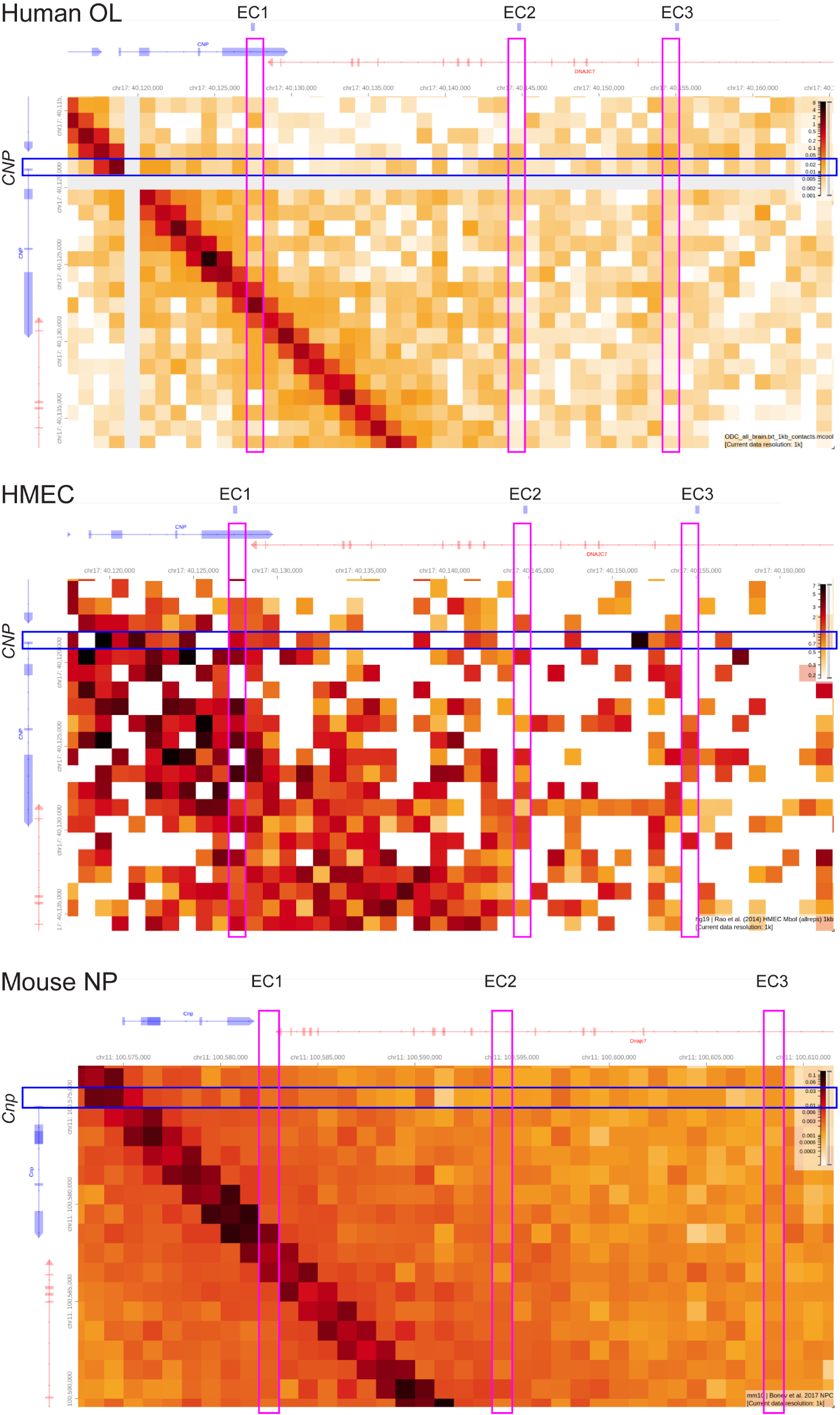


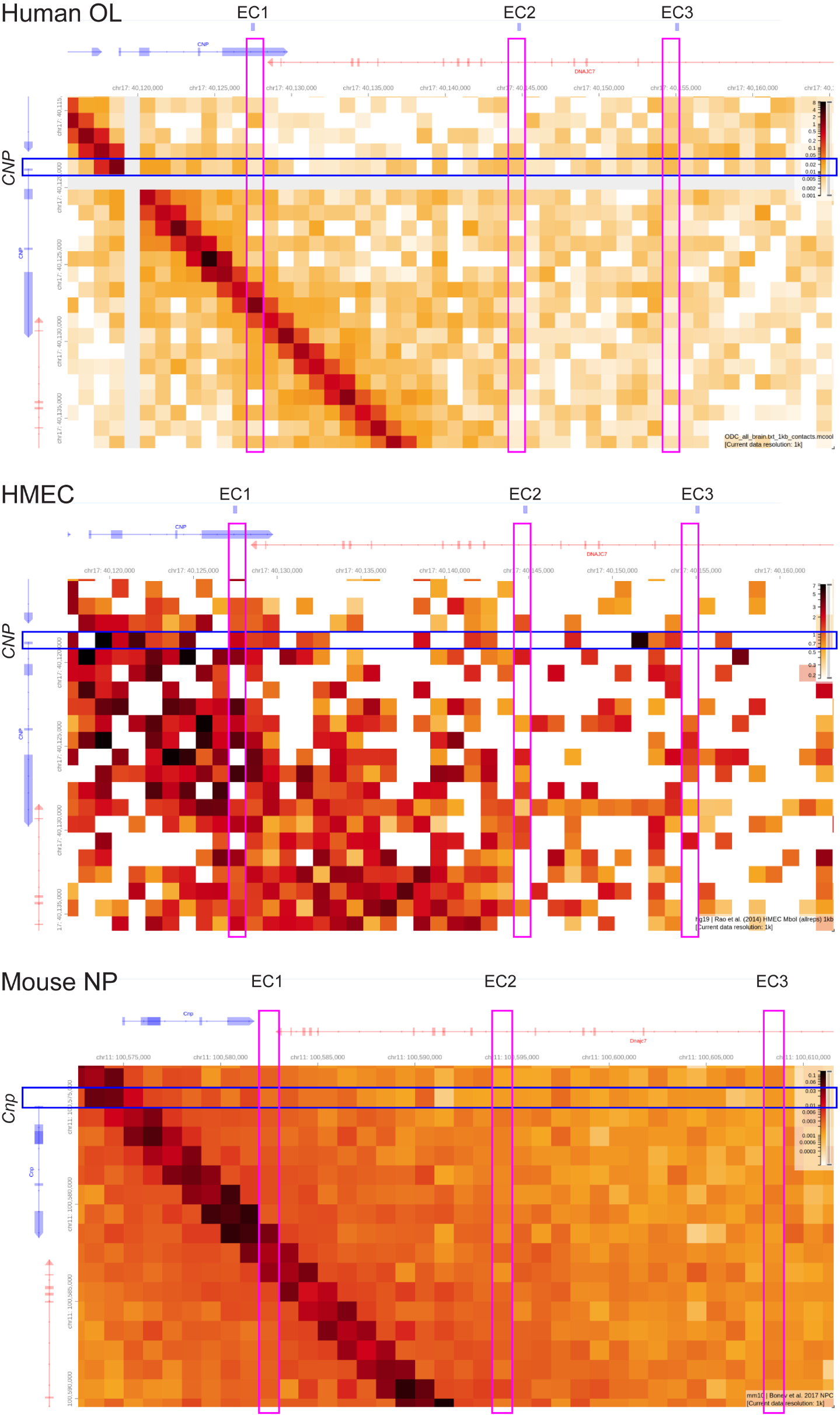


**
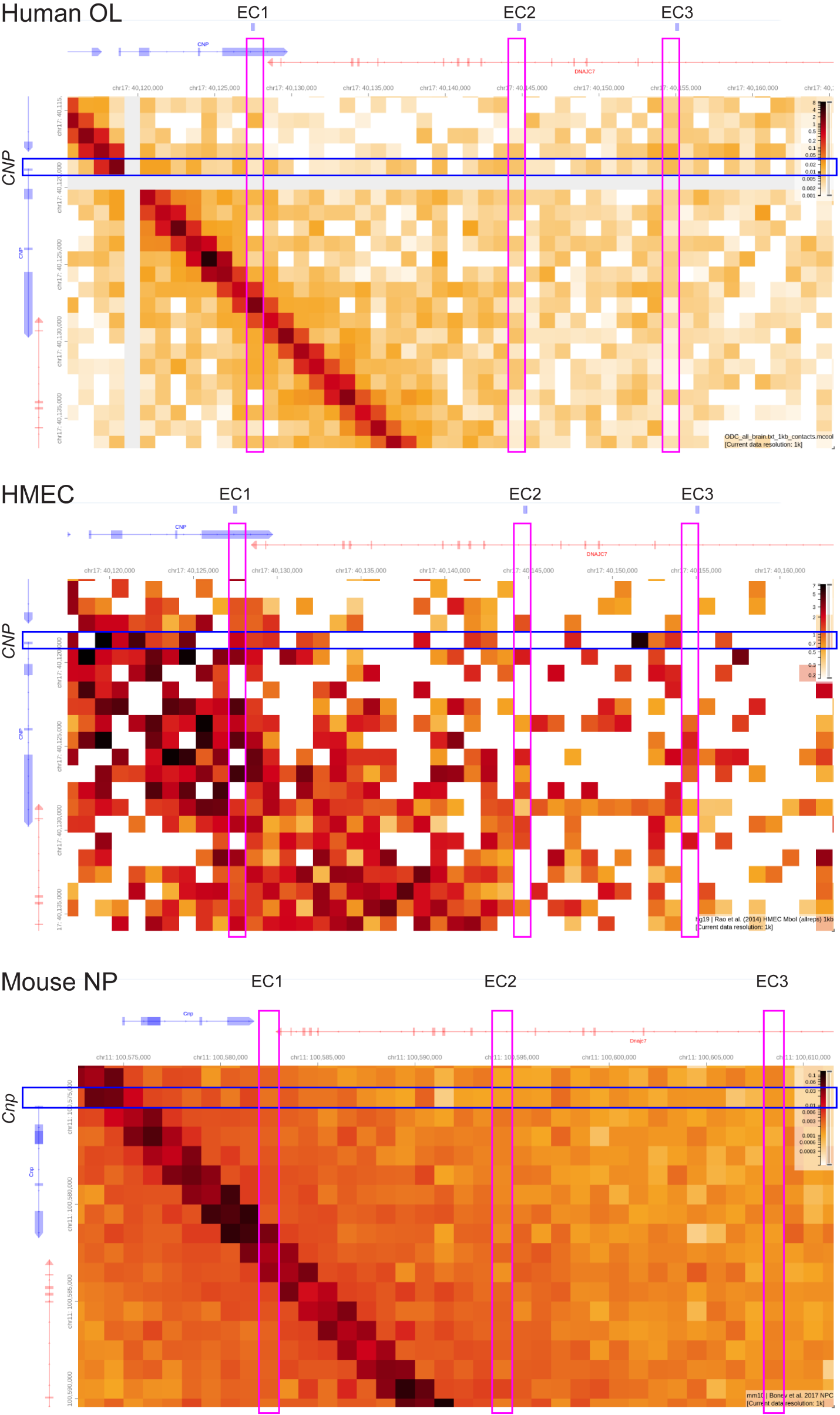
**

**Figure S3. Zoomed in images for Figure 4D of the main manuscript**

**
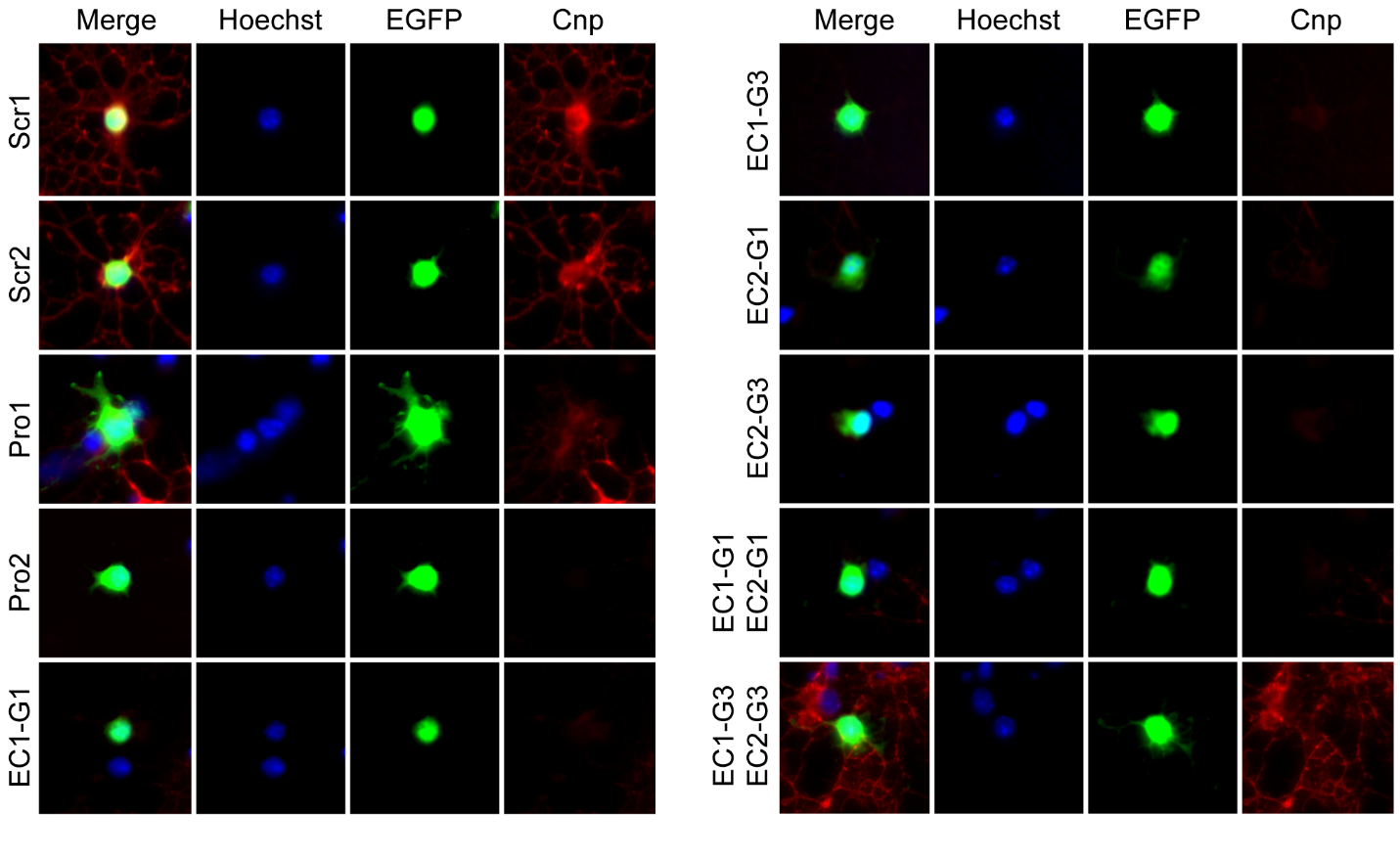
**

**Figure S4. The NIH Roadmap Epigenomics Project H3K27ac ChIP-seq data for Cnp-E1 and Cnp-E2**

**
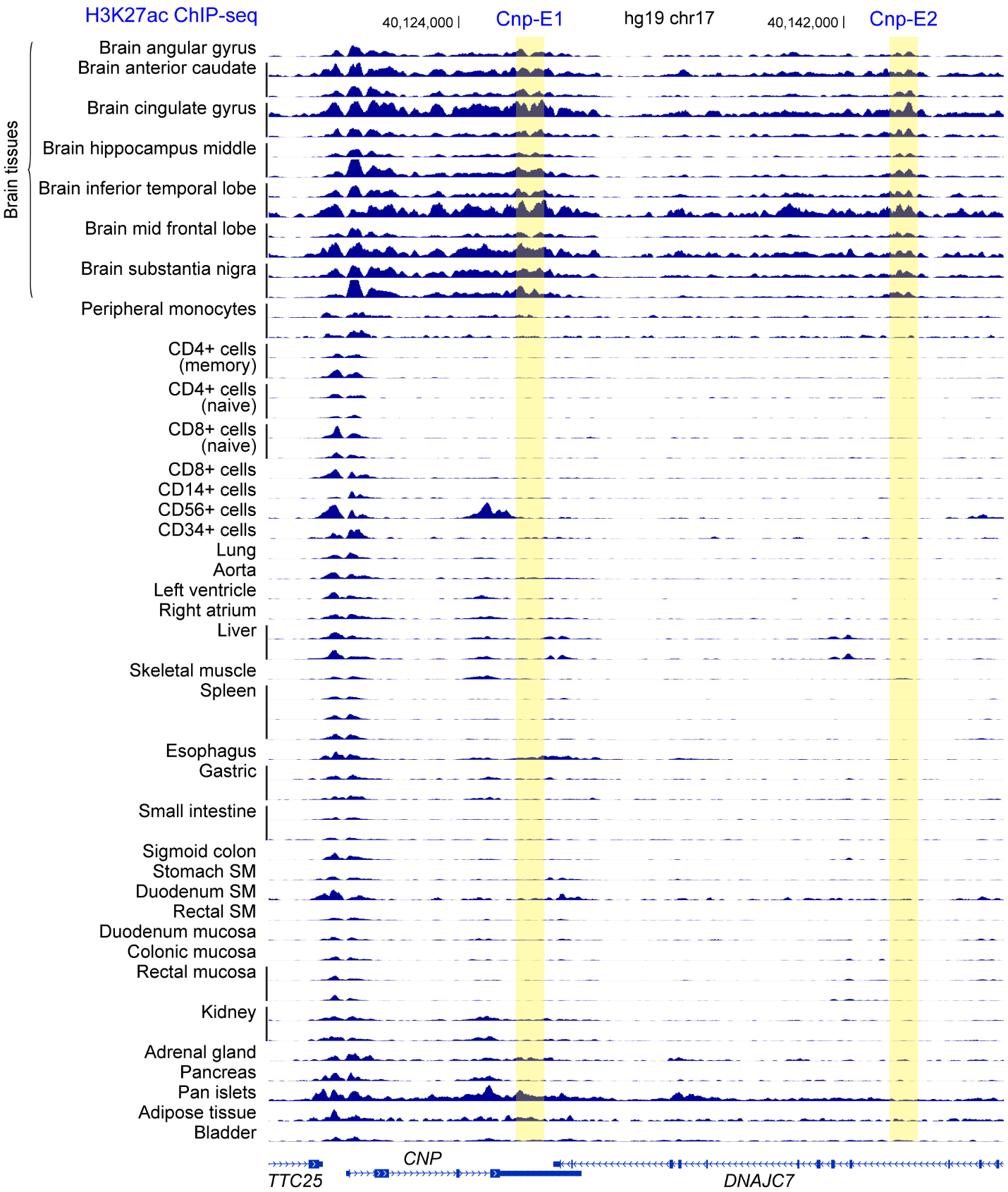
**

**Table S1. Guide RNA sequences**

| Guide RNA | Sequence |
| --- | --- |
| Scr1 | GCACTACCAGAGCTAACTCA |
| Scr2 | TGCGAATACGCCCACGCGAT |
| Pro1 | GGGGGACCGCTTGTCAGTTG |
| Pro2 | TGCTCCCCCTCAGAACACCT |
| Pro3 | AAATCCACCATTCCCCTCCG |
| Pro4 | CTACCTAACCTCTTAGTCTC |
| EC1-G1 | TGTACGCAGCCCTAACCCCA |
| EC1-G2 | TGGCTTCAGACACATAGAGA |
| EC1-G3 | GGACCGATGGCCCCAAGCTG |
| EC1-G4 | AGCTGTGACGAGGCTGGGTA |
| EC2-G1 | AGCAGTTCTAACTACTGTCA |
| EC2-G2 | ACATCACCATGAGCTACAGT |
| EC2-G3 | GAGGATTCTGTCACAAACTC |
| EC2-G4 | CAAATGAAGAATTATGATCC |
| EC3-G1 | CTGGCCCGATATGCAATGTG |
| EC3-G2 | TTCTAAGATGCAAGACCCAG |
| EC3-G3 | TTCACAGAAACGCGGCTGAT |
| EC3-G4 | GGAGGCAAAGTCCATCCCCT |
| NC1-G1 | ACTGTCCGGCTCCTATAGCA |
| NC1-G2 | GCTTGAGGGATATGGACAAG |
| NC1-G3 | ATTCTGCTCCTGCCTGCCAG |
| NC1-G4 | CCTCAGCCCTCTCCAGTGCT |
| NC2-G1 | GCCCTCAAACATGCTAGGCA |
| NC2-G2 | TAGCTGGCACTCTACAAATG |
| NC2-G3 | TCTGTATCCCCACACTAAAG |
| NC2-G4 | CTACTGCATCTGTGTACGCT |
| NC3-G1 | TCCAATGAGTGGATAGTCTG |
| NC3-G2 | GTCTATGTAGCCTTCCATGG |
| NC3-G3 | CCCAAGACTCAACTACCTGT |
| NC3-G4 | TGGAACTCACTTCATCCATC |

**Table S2. High-confidence motif matches in Cnp-E1 and Cnp-E2, as determined by FIMO and HOCOMOCO**

>Cnp-EC1 (mm10 chr11:100581624-100582086)

TGGGTTGTCACCCGAGAATCTGGGATGTATGCTCCTACCCTCACCCTGTGTTGCCCACCTCAGCCACCACTAAGACTGATACTGAAATAAATCATGTTAATCCCAGCTGTGTGCTATCACTCTTGGCCTTCTATGTCCAGCCCAGTCTTCCCTGGGGTTAGGGCTGCGTACATTAAGACCAGCTTTGTCCCACAGCTTGGGGCCATCGGTCCTGTCTCCCTTCTGCTGGCTGGGCACAGCTGTGACGAGGCTGGGTAGGGCAACAGTGTTGGCTTTCATGAAGCAGAAAGGAGAGGCTGGCTTCAGACACATAGAGAAGGTGGGGATTGGCTGGCAGCCAGAAACTTGGAGCACAAAGCCTTTATCTGACAGCTAAGGCAACCGACCACATGCAGGTGGCCATGTCCCAAAGACCTCTCAGCAAGGCCCAGAGTTCCCAGCCTGGGCTTTGTGGGGTACTTGG

| Start position | End position | HOCOMOCO ID | *q* value |
| --- | --- | --- | --- |
| 34 | 50 | SALL1_MOUSE.H11MO.0.D | 0.0061 |
| 34 | 46 | SPIC_HUMAN.H11MO.0.D | 0.0078 |
| 39 | 58 | ZN121_HUMAN.H11MO.0.C | 0.00758 |
| 52 | 70 | TBX15_HUMAN.H11MO.0.D | 0.00271 |
| 53 | 69 | SALL1_MOUSE.H11MO.0.D | 0.00464 |
| 81 | 95 | HXC6_HUMAN.H11MO.0.D | 0.00344 |
| 81 | 95 | HXC6_MOUSE.H11MO.0.D | 0.00344 |
| 84 | 95 | MEIS1_MOUSE.H11MO.0.A | 0.0067 |
| 84 | 95 | MEIS1_HUMAN.H11MO.0.A | 0.0067 |
| 85 | 95 | HXA9_MOUSE.H11MO.0.B | 0.00843 |
| 85 | 95 | HXA9_HUMAN.H11MO.0.B | 0.00843 |
| 96 | 105 | PITX2_HUMAN.H11MO.0.D | 0.00641 |
| 96 | 105 | PITX2_MOUSE.H11MO.0.D | 0.00639 |
| 103 | 120 | PTF1A_HUMAN.H11MO.0.B | 0.00726 |
| 103 | 112 | TFAP4_HUMAN.H11MO.0.A | 0.00264 |
| 103 | 120 | PTF1A_MOUSE.H11MO.0.A | 0.00726 |
| 103 | 116 | LYL1_HUMAN.H11MO.0.A | 0.00685 |
| 103 | 116 | LYL1_MOUSE.H11MO.0.A | 0.00684 |
| 148 | 157 | PRD16_MOUSE.H11MO.0.B | 0.00126 |
| 222 | 241 | ZN331_HUMAN.H11MO.0.C | 0.00805 |
| 227 | 237 | ZN449_HUMAN.H11MO.0.C | 0.00551 |
| 228 | 247 | ZN331_HUMAN.H11MO.0.C | 0.000219 |
| 230 | 243 | LYL1_MOUSE.H11MO.0.A | 0.00684 |
| 230 | 243 | LYL1_HUMAN.H11MO.0.A | 0.00685 |
| 247 | 261 | KLF4_MOUSE.H11MO.0.A | 0.00402 |
| 247 | 261 | KLF5_MOUSE.H11MO.0.A | 0.00427 |
| 285 | 307 | Z324A_HUMAN.H11MO.0.C | 0.000514 |
| 312 | 326 | KLF4_MOUSE.H11MO.0.A | 0.00831 |
| 314 | 330 | SALL1_MOUSE.H11MO.0.D | 0.00696 |
| 319 | 331 | MZF1_HUMAN.H11MO.0.B | 0.00114 |
| 326 | 340 | NFIC_MOUSE.H11MO.1.A | 0.00965 |
| 326 | 342 | NFIC_HUMAN.H11MO.0.A | 0.000706 |
| 327 | 341 | NFIA_HUMAN.H11MO.0.C | 0.000255 |
| 327 | 341 | NFIA_MOUSE.H11MO.0.C | 0.000255 |
| 328 | 342 | NFIC_MOUSE.H11MO.1.A | 0.000483 |
| 349 | 359 | SOX9_MOUSE.H11MO.1.A | 0.00888 |
| 414 | 433 | ZN331_HUMAN.H11MO.0.C | 0.00871 |
| 440 | 459 | ZN331_HUMAN.H11MO.0.C | 0.006 |

>Cnp-EC2 (mm10 chr11:100594705-100595099)

GAGGTACAACGAGGAAAAGCCAGGGACAGCGGAGTAGCCACGCCCTTGGCCTCACAAGCTCAACGTTCTATGCAGAGCTGGCTCTGCTCATCGTGAGGAACATCAGTTGAGGCTCAGCAATGCTTACTGCCACCCCCAACCACGTCCCCAGTAGGGACAGCTGCAGGCAAAGAAGATCTTTGTTTCTTCTAAGTTCATCTGTTTCCCCTGCCAGGATCATAATTCTTCATTTGTGACATCACCATGAGCTACAGTGGGGGGGCTGGGATGTCACAAACAGAGGATTCTGTCACAAACTCAGGATTCTGGCTCCAGAGTGAGCAAGAGAGAAAAGAAACCCAAAAGACTGCCTTTCAGGACAAAGCCGTGACAGTAGTTAGAACTGCTTTCCAAAC

| Start position | End position | HOCOMOCO ID | *q* value |
| --- | --- | --- | --- |
| 11 | 32 | PATZ1_HUMAN.H11MO.0.C | 0.00862 |
| 33 | 46 | KLF1_HUMAN.H11MO.0.A | 0.00732 |
| 33 | 51 | KLF3_HUMAN.H11MO.0.B | 0.00239 |
| 33 | 46 | KLF1_MOUSE.H11MO.0.A | 0.00769 |
| 33 | 51 | KLF3_MOUSE.H11MO.0.A | 0.00237 |
| 36 | 50 | KLF9_HUMAN.H11MO.0.C | 0.00533 |
| 37 | 46 | KLF4_HUMAN.H11MO.0.A | 0.00037 |
| 37 | 47 | KLF12_HUMAN.H11MO.0.C | 0.00167 |
| 37 | 51 | KLF4_MOUSE.H11MO.0.A | 0.00232 |
| 37 | 51 | KLF5_MOUSE.H11MO.0.A | 0.00152 |
| 41 | 53 | NR6A1_MOUSE.H11MO.0.D | 0.0091 |
| 41 | 61 | RXRA_MOUSE.H11MO.0.A | 0.00729 |
| 46 | 64 | ZN667_HUMAN.H11MO.0.C | 0.000445 |
| 79 | 87 | ZN554_HUMAN.H11MO.1.D | 0.00195 |
| 127 | 143 | EGR1_HUMAN.H11MO.0.A | 0.00696 |
| 129 | 150 | RREB1_MOUSE.H11MO.0.D | 0.00965 |
| 129 | 150 | SP1_HUMAN.H11MO.0.A | 0.00338 |
| 129 | 150 | RREB1_HUMAN.H11MO.0.D | 0.00965 |
| 131 | 149 | KLF16_HUMAN.H11MO.0.D | 0.0004 |
| 132 | 153 | RREB1_MOUSE.H11MO.0.D | 0.00965 |
| 132 | 153 | RREB1_HUMAN.H11MO.0.D | 0.00965 |
| 133 | 149 | EGR1_HUMAN.H11MO.0.A | 0.00696 |
| 150 | 164 | MYOD1_HUMAN.H11MO.0.A | 0.000837 |
| 150 | 164 | MYOD1_MOUSE.H11MO.0.A | 0.000341 |
| 153 | 166 | ASCL1_MOUSE.H11MO.0.A | 0.00112 |
| 153 | 166 | ASCL1_HUMAN.H11MO.0.A | 0.00435 |
| 154 | 166 | MYOG_HUMAN.H11MO.0.B | 4.22E-05 |
| 154 | 166 | MYOG_MOUSE.H11MO.0.A | 4.22E-05 |
| 154 | 164 | MYOD1_MOUSE.H11MO.1.A | 9.67E-05 |
| 154 | 164 | MYF5_MOUSE.H11MO.0.D | 0.000349 |
| 156 | 166 | TFE2_MOUSE.H11MO.0.A | 0.00056 |
| 156 | 166 | MYOD1_HUMAN.H11MO.1.A | 0.000118 |
| 156 | 165 | ITF2_HUMAN.H11MO.0.C | 0.00887 |
| 156 | 166 | TFE2_HUMAN.H11MO.0.A | 0.000558 |
| 157 | 177 | ZN563_HUMAN.H11MO.0.C | 0.00341 |
| 169 | 184 | SOX10_MOUSE.H11MO.0.B | 0.0021 |
| 169 | 184 | SOX10_HUMAN.H11MO.0.B | 0.0021 |
| 170 | 185 | SOX9_MOUSE.H11MO.0.A | 0.00508 |
| 170 | 185 | SOX9_HUMAN.H11MO.0.B | 0.00508 |
| 178 | 197 | ZFP28_HUMAN.H11MO.0.C | 0.00132 |
| 195 | 208 | LYL1_MOUSE.H11MO.0.A | 0.00485 |
| 195 | 208 | LYL1_HUMAN.H11MO.0.A | 0.00485 |
| 198 | 212 | FOXD3_HUMAN.H11MO.0.D | 0.008 |
| 198 | 212 | FOXD3_MOUSE.H11MO.0.C | 0.00796 |
| 207 | 215 | NFIB_MOUSE.H11MO.0.C | 0.00541 |
| 208 | 216 | NFIB_HUMAN.H11MO.0.D | 0.00372 |
| 208 | 216 | NFIC_HUMAN.H11MO.1.A | 0.00444 |
| 208 | 216 | NFIC_MOUSE.H11MO.0.A | 0.00444 |
| 228 | 247 | ATF2_HUMAN.H11MO.1.B | 0.00359 |
| 233 | 243 | ATF7_MOUSE.H11MO.0.D | 0.000972 |
| 233 | 243 | ATF2_MOUSE.H11MO.0.A | 0.00196 |
| 233 | 243 | ATF2_HUMAN.H11MO.0.B | 0.00196 |
| 249 | 263 | ZIC4_HUMAN.H11MO.0.D | 0.0051 |
| 252 | 270 | ZBT17_MOUSE.H11MO.0.A | 0.0077 |
| 252 | 270 | ZBT17_HUMAN.H11MO.0.A | 0.00122 |
| 252 | 268 | EGR1_HUMAN.H11MO.0.A | 0.00696 |
| 252 | 264 | GLIS1_HUMAN.H11MO.0.D | 0.00825 |
| 253 | 274 | PATZ1_HUMAN.H11MO.0.C | 0.00737 |
| 253 | 266 | ZN740_HUMAN.H11MO.0.D | 0.000601 |
| 253 | 267 | ZN281_MOUSE.H11MO.0.A | 0.00211 |
| 254 | 268 | ZN281_HUMAN.H11MO.0.A | 0.00193 |
| 258 | 267 | SALL4_MOUSE.H11MO.0.A | 0.00836 |
| 258 | 267 | SALL4_HUMAN.H11MO.0.B | 0.00836 |
| 323 | 342 | ZN274_HUMAN.H11MO.0.A | 0.005 |
| 324 | 342 | ZIM3_HUMAN.H11MO.0.C | 0.00284 |
| 326 | 347 | STAT1_MOUSE.H11MO.0.A | 0.0007 |
| 326 | 344 | STAT1_HUMAN.H11MO.1.A | 0.000696 |
| 326 | 344 | STAT2_MOUSE.H11MO.0.A | 0.000376 |
| 326 | 344 | STAT2_HUMAN.H11MO.0.A | 0.000375 |
